# Repeated *Porphyromonas gingivalis* W83 exposure leads to release pro-inflammatory cytokynes and angiotensin II in coronary artery endothelial cells

**DOI:** 10.1038/s41598-019-54259-y

**Published:** 2019-12-18

**Authors:** Sergio M. Viafara-García, Sandra Johanna Morantes, Yersson Chacon-Quintero, Diana Marcela Castillo, Gloria Inés Lafaurie, Diana Marcela Buitrago

**Affiliations:** Unit of Basic Oral Investigation-UIBO, School of Dentistry, Universidad El Bosque, Bogota, Colombia

**Keywords:** Hypertension, Periodontitis, Infection

## Abstract

The role of *Porphyromonas gingivalis* (*P. gingivalis*) or its virulence factors, including lipopolysaccharide (LPS) not only has been related with periodontitis but also with endothelial dysfunction, a key mechanism involved in the genesis of atherosclerosis and hypertension that involving systemic inflammatory markers as angiotensin II (Ang II) and cytokines. This study compares the effect of repeated and unique exposures of *P. gingivalis* W83 LPS and live bacteria on the production and expression of inflammatory mediators and vasoconstrictor molecules with Ang II. Human coronary artery endothelial cells (HCAEC) were stimulated with purified LPS of *P. gingivalis* (1.0, 3.5 or 7.0 μg/mL) or serial dilutions of live bacteria (MOI 1: 100 - 1:0,1) at a single or repeated exposure for a time of 24 h. mRNA expression levels of AGTR1, AGTR2, IL-8, IL-1β and MCP-1 were determined by RT-qPCR, and IL-6, MCP-1, IL-8, IL-1β and GM-CSF levels were measured by flow cytometry, ELISA determined Ang II levels. Live bacteria in a single dose increased mRNA levels of AGTR1, and repeated doses increased mRNA levels of IL-8 and IL-1β (p < 0.05). Repeated exposure of live-*P. gingivalis* induced significant production IL-6, MCP-1 and GM-CSF (p < 0.05). Moreover, these MCP-1, IL-6 and GM-CSF levels were greater than in cells treated with single exposure (p < 0.05), The expression of AGTR1 and production of Ang II induced by live-*P. gingivalis* W83 showed a vasomotor effect of whole bacteria in HCAEC more than LPS. In conclusion, the findings of this study suggest that repeated exposure of *P. gingivalis* in HCAEC induces the activation of proinflammatory and vasoconstrictor molecules that lead to endothelial dysfunction being a key mechanism of the onset and progression of arterial hypertension and atherosclerosis.

## Introduction

Inflammation has received much attention as an determining factor in hypertension and atherosclerosis progression^1,2^. Although the effect of inflammation on endothelial dysfunction has been widely studied, the mechanisms of inflammation in hypertension have not been completely elucidated^2^.

Chronic systemic inflammation induced by periodontitis is linked to endothelial dysfunction due to the entry of Gram-negative anaerobes into the bloodstream after various stimuli, such as tooth brushing and chewing^3^ and periodontal treatment^3–5^ likewise, DNA^6,7^ of periodontal pathogens (e.g., *Porphyromonas gingivalis*) and live bacteria^8,9^ have been demonstrated in atherosclerotic coronary lesions^10,11^, and increased systemic inflammatory markers induced by endotoxemia have been associated with hypertension/early atherosclerosis and periodontal disease^12,13^. Hence, there is a potential association between vascular inflammation and cardiovascular disease (CVD), since endothelial cells are the primary targets of immunological attack in inflammatory responses^14^.

The endothelium has been described as a secretory unit of pathogen-associated molecular patterns (PAMPs) that generates secreting soluble mediators. Cytokines and chemokines, as well as other products such as reactive oxygen species and metalloproteinases (MMPs) have been correlated with greater atheromatous lipid core increases in the presence of monocytes-macrophages and in the levels of IL-8, MCP-1, MMP-8 and MMP-9 in atherosclerotic plaques^15,16^. *P*. *gingivalis*, a major periodontal pathogen in periodontitis, has been shown to stimulate cytokine/chemokine production, which induces expression of cell adhesion molecules, including intercellular adhesion molecule (ICAM-1), vascular cell adhesion molecule (VCAM-1) and p-selectin, which are considered key steps in the onset of endothelial dysfunction^17,18^. *P*. *gingivalis* also activates endothelial cells, triggers smooth muscle cell proliferation and therefore impairs vasomotor function^19,20^.

The evolving role of Ang II as a regulator of endothelial cell function and its action by stimulating various receptors, specifically angiotensin II type 1 receptor (AGTR1) stimulates oxidative stress, fibrosis, cell proliferation and a release of cytokines and chemokines which in turn mediates tissue inflammation^21,22^. Recently, the evidence has indicated that low-grade inflammation may be involved in the development of hypertension^23^. In fact, the initiation or progression of periodontitis might involve a local renin-angiotensin system (RAS) activation^21,22^, that could lead to an increase in blood pressure, a decrease in nitric oxide, inflammation and development of atherosclerosis and endothelial dysfunction^22,24,25^.

Several bacteraemia episodes occur continuously in patients with periodontitis and are even induced by daily dental-care activities (i.e., brushing, flossing and chewing)^26,27^. Repeated episodes are also generated during intensive periodontal treatments that could induce an inflammatory acute response in the endothelium^28,29^. Although many reports examining *P. gingivalis* have demonstrated vascular endothelium activation through the selective recruitment of leukocytes to inflammatory foci in HCAEC via Toll-like receptor 2 (TLR2) after a single exposure to endotoxin^16,30,31^, it has been not studied in an *in-vitro* approach using repeated exposure in endothelial models of inflammatory responses. Therefore, these *in-vitro* cell models do not represent or mimic the vascular pathophysiology in patients with periodontitis, since the endothelium is exposed to repeated and accumulative doses of transient endotoxemia that could represent changes in the response, compared to the single exposure models. The purpose of this study was to compare the inflammatory and vasoactive response of Ang II of HCAEC stimulated with single and repeated exposure of lipopolysaccharide (*P. gingivalis*-LPS) and live-*P. gingivalis* W83.

## Results

### Effect of *P. gingivalis* on the expression of AGTR1, AGTR2, IL-8, IL-1β, MCP-1 in HCAEC

**Figure 1:**
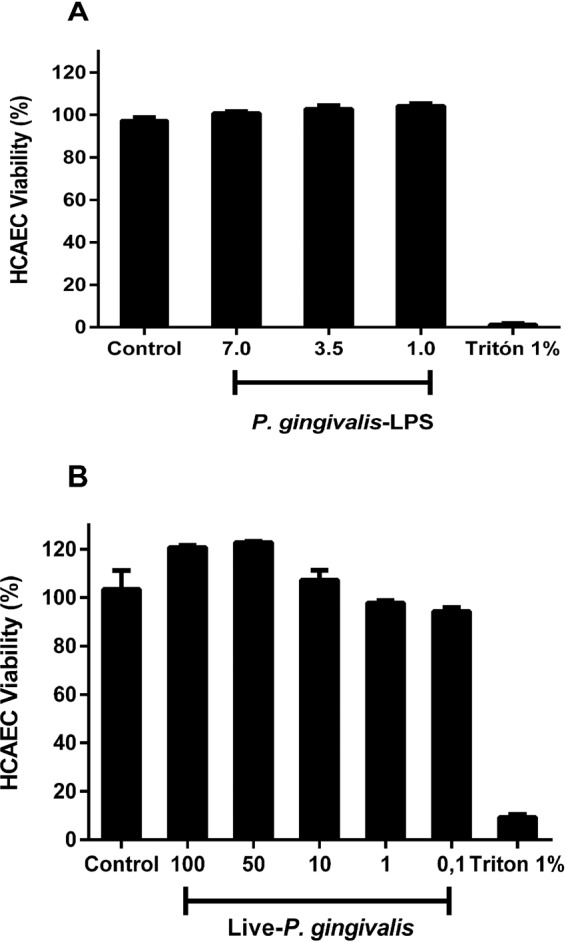
Viability of HCAECs after repeated treatments with live-*P. gingivalis* (**A**) and ***P****. gingivalis*-LPS (**B**). The HCAECs were stimulated to repeated live-*P. gingivalis* (MOI 1:100 - 1:0,1) and *P. gingivalis*-LPS (1.0, 3.5 and 7.0 µg/mL) exposures, during 24 h. Cell viability was determined according to the fluorometric detection after reduction of resazurin in the resorufin product using AlamarBlue. 1% was considered our positive control of cell death. Percentage of cell viability with respect to the control. *Represents the statistical difference with respect to the control or without stimulus. (p < 0.05). Three independent experiments were performed; the results are presented as the means ± SEM (n = 3).

The biochemical characterization to confirm the degree of purity of LPS of *P. gingivalis* W83 showed a semi-rough LPS chemotype with bands of low molecular weight free of contaminants such as nucleic acids and proteins high endotoxic activity at very low concentrations similar to observed by commercial LPS (*P. gingivalis* ATCC 33277, InvivoGen)^32^. In relation to cell viability, our results demonstrated that LPS and live bacteria did not affect HCAECs (Fig. 1).

**Figure 2:**
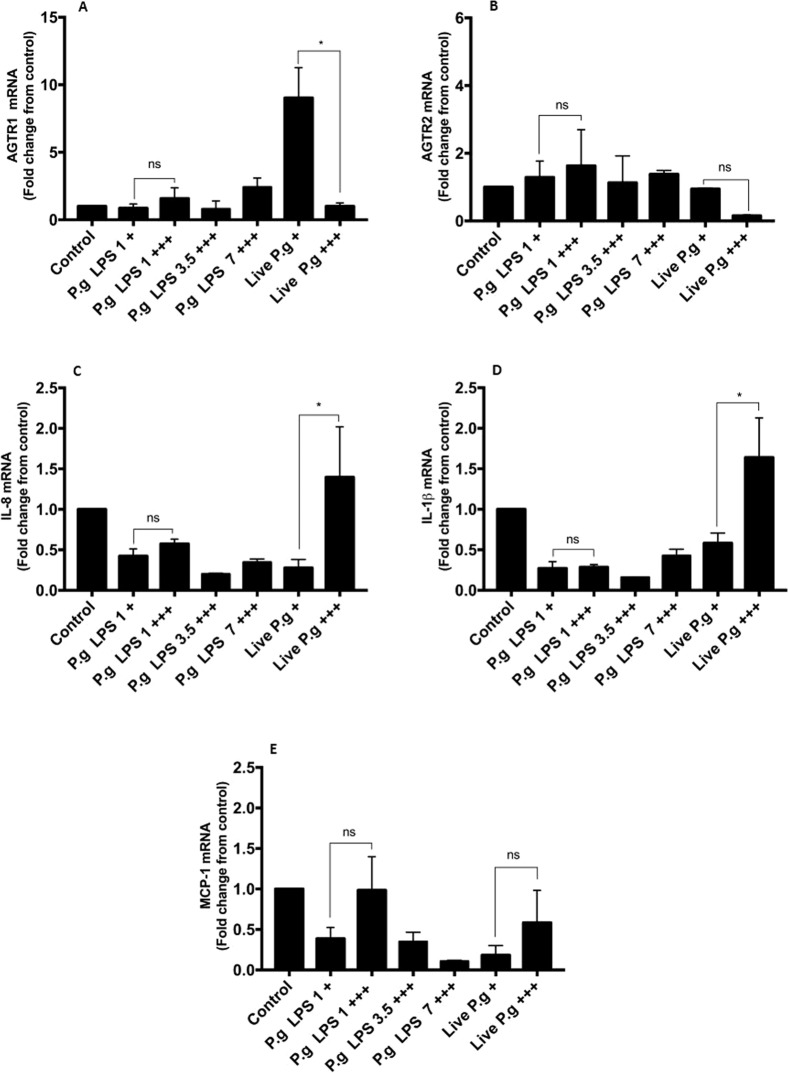
mRNA expression levels in HCAEC stimulated with *P. gingivalis*-LPS or live-*P. gingivalis*. Monolayers of HCAEC cultured in 12-well plates were stimulated with *P. gingivalis*-LPS (1.0, 3.5, 7.0 µg/mL) or serial  dilutions of *P. gingivalis* (MOI 1:100- 1:0,1) for 24 h under repeated exposure (+++) or single exposure (+). After stimulation, (**A**) AGTR1, (**B**) AGTR2, (**C**) IL-8, (**D**) IL-1β, (**E**) MCP-1, mRNA levels were measured by are expressed as the means by RT-qPCR. Results are expressed as the means ± SEM (n=3). Statiscal significance is represented as *p < 0.05, and (ns) for not significant.

In this study, we evaluated the expression of proinflammatory cytokine mRNA and AGTR genes in HCAEC stimulated with *P. gingivalis*-LPS and live-*P. gingivalis W83*. The cells stimulated at single and repeated exposure with the different concentrations of *P. gingivalis*-LPS did not show significant changes for any of the pro-inflammatory markers even in comparison with the control group (Fig. 2), while repeated exposure of MOI 1:100 live-*P. gingivalis* compared to single exposure to the same MOI 1:100, significantly affects the expression of IL-8 and IL-1β (p < 0.05). In contrast, the expression of MCP-1 was not significantly affected by the treatments; however, an apparent reduction in its mRNA was observed after single exposure to LPS and live bacteria. (Fig. 2A–E). On the other hand, a single exposure to live-*P. gingivalis* increased the AGTR1 expression (Fig. 2A) compared to unchallenged HCAEC and challenged with live bacteria to a repeated exposure (p < 0.05), whereas AGTR2 (Fig. 2B) It was not because of the results of the treatments evaluated.

### *P. gingivalis* induces IL-1β, IL-8, IL-6, MCP-1, and GM-CSF production in HCAEC

In a pilot test, HCAEC cells were stimulated at day 1, day 3, and day 5; the supernatant was removed each 48 h to reaching a total of 7 days under repeated exposure. In general, we found similar results in a longer time frame and shorter time frame to induce the production of IL8 in the majority of LPS concentrations assessed (Supplementary Fig. 1). However, in a short period of time an overproduction of IL8 was achieved, suggesting greater endothelial activation than with a long period of time in which endothelial activation appears to decrease. This model could be used to study endotoxin tolerance of periodontal pathogens, an aspect that requires further research. Similarly, when comparing IL6 and IL1, there were also no modifications between the 24 h and 7 day in repeated exposure models (Supplementary Fig. 2).

**Figure 3:**
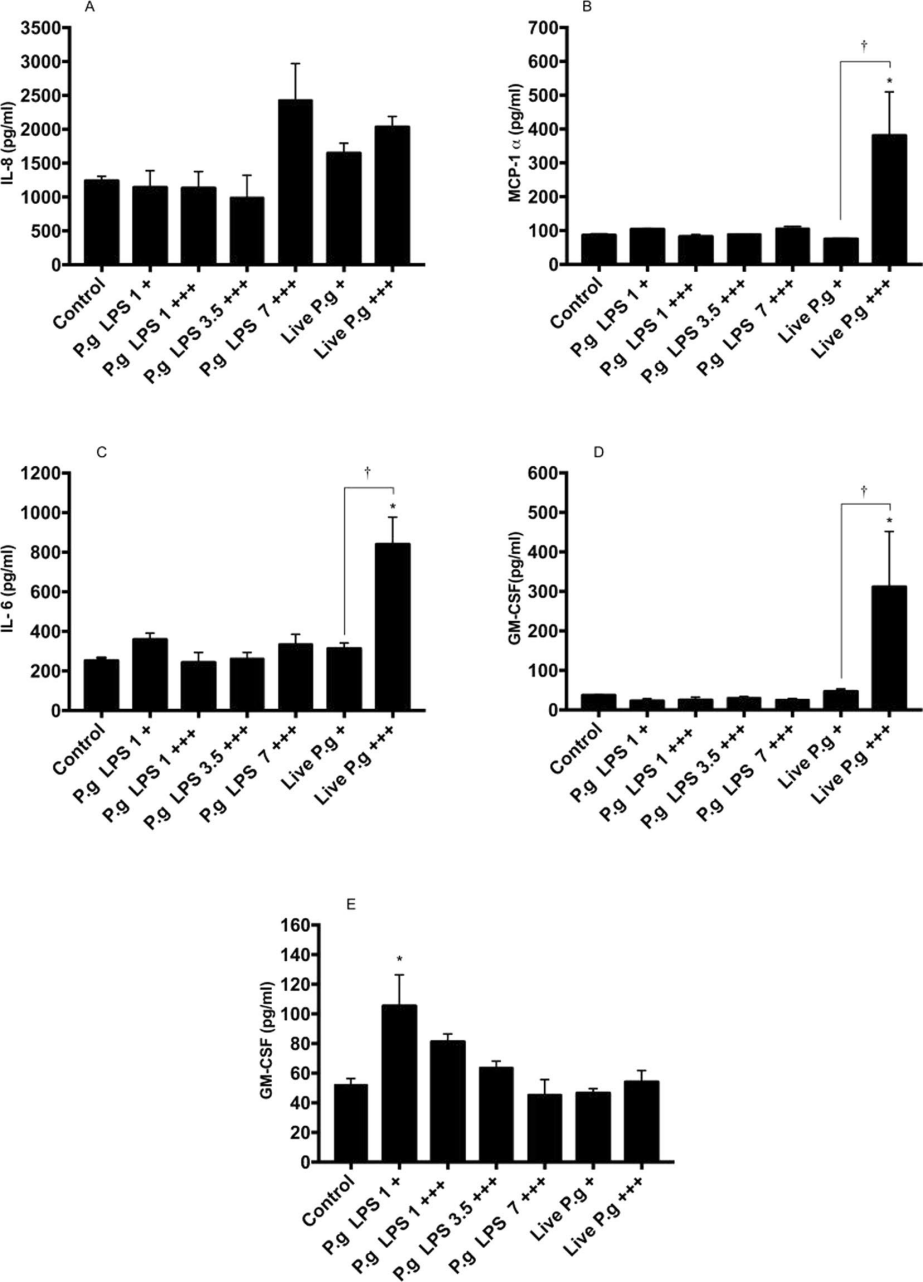
Chemokines and cytokines secreted in HCAEC stimulated with *P. gingivalis*-LPS or live-*P. gingivalis*. Monolayers of HCAEC cultured in 12-well plates were stimulated with *P. gingivalis*-LPS (1.0, 3.5, 7.0 µg/mL) or *P. gingivalis* (MOI 1:100 - 1:0,1) for 24 h under repeated exposure (+++) or single exposure (+). After stimulation, levels of the following chemokines were measured in cell culture supernatants using a cytometric bead array: (**A**) IL-8, (**B**) MCP-1, (**C**) IL-6, (**D**) GM-CSF, (**E**) IL-1β. Symbol (*) means p < 0.05 vs control cells; (**) means p < 0.01 vs control cells; ^†^p < 0.05 compared vs cells treated by single exposure; ^††^p < 0.01 compared vs cells treated by single exposure; (ns) for not significant. Three independent experiments were performed; the results are presented as the means ± SEM (n = 3).

The HCAECs were stimulated with *P. gingivalis*-LPS or live-*P. gingivalis* for 24 h by repeated or single exposures to evaluate the levels of IL-8, MCP-1, IL-6, IL-1β and GM-CSF in culture supernatants. Repeated exposure to live-*P. gingivalis* induced a significant increase in the production of IL-6, MCP-1 and GM-CSF, compared to the control group (p < 0.05), while the *P. gingivalis*-LPS is a single exposure only induced change in IL-1β (Fig. 3). The chemokine IL-8 was increased mainly in HCAEC challenged with live-*P. gingivalis* and *P. gingivalis*-LPS at 7 μg/mL at repeated expositions, but not significantly compared to the control (Fig. 3A). After considering the results so far, endothelial responses differed between approaches. HCAEC to repeated exhibitions with live-*P. gingivalis* achieved a significantly higher production of MCP-1, IL-6 and GM-CSF than treated endothelial cells at a single exposure of bacteria (Fig. 3B–D), while producing IL-1β (Fig. 3E) was higher in HCAEC challenged with LPS at a single exposure of 1μg/mL (p < 0.05).

### *P. gingivalis* increases angiotensin II levels in HCAEC

**Figure 4:**
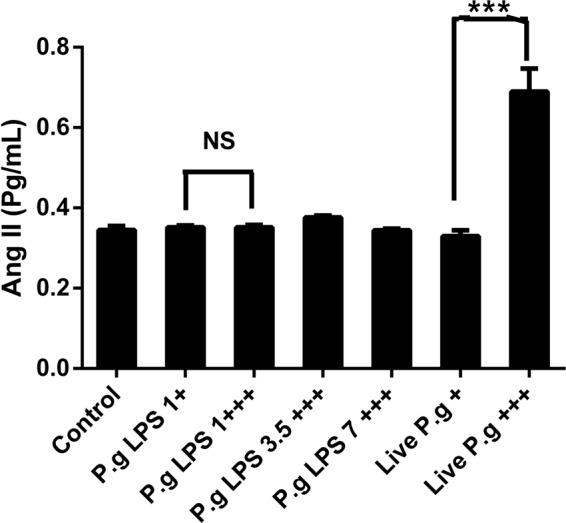
Angiotensin II levels are determined in the HCAEC  cell culture supernatant stimulated to single (+) or repetitive (+++) exposures of *P. gingivalis*-LPS or live-*P*. *gingivalis* by the ELISA kit. The results are expressed as the means ± SEM (n=3)  with a statistical significance represented as (*)p < 0.05 vs. control, (^†^)p < 0.05 compared vs cells treated by single exposure; (ns) for not significant.

Ang II concentration was evaluated in HCAEC supernatants after *P. gingivalis*-LPS stimulation and live-*P. gingivalis* in single and repeated expositions. Only live-*P. gingivalis* (MOI 1:100) at repeated exposure significantly increased the concentration of Ang II, compared to the live bacteria single exposure (1.9 times) and the control cells (2.1 times) (Fig. 4).

## Discussion

Oral pathogens associated with periodontitis, such as *P. gingivalis*, have been of particular interest due to the high levels of bacteraemia and endotoxemia after routine dental procedures and everyday oral activities, such as tooth brushing^7^. The biological mechanisms underlying the potential link between periodontitis and atherosclerosis or hypertension remain unclear, mainly in terms of inflammatory and vasoactive endothelial responses to *P. gingivalis* with repeated exposures. In this study, we evaluated the effects of single versus repeated administration of *P. gingivalis*-LPS and whole bacteria on the pro-inflammatory mediators and vasoactive markers involved in HCAEC atherosclerotic response.

In relation to cell viability, there are no reports in the literature that demonstrate the *in vitro* effect of periodontopathogens such as *P. gingivalis* on HCAEC cells at repeated exposures and at concentrations greater than 2 mg/mL with LPS^33,34^. Our results demonstrated that of *P. gingivalis*-LPS and the bacteria did not affect the cellular viability of HCAEC at the concentrations evaluated; data similar to those reported by Chou *et al*.^35^ where even 100 µg/mL of *P. gingivalis*-LPS W83 on leukocytes as PMN did not affect its viability^36^. However, depending on the type of cell line differences may occur, in this way have been described in esophageal cell lines as OE19 (adenocarcinoma), OE21 (squamous cell carcinoma), a sensitivity different from that reported in oral tumor cells (HN30), where the esophageal cancer cells were only sensitive to LPS *P. gingivalis* W83 concentrations of 20, 50 and 100 μg/mL (24,48 and 72 h), unlike oral cells (HN30) where the LPS increases the viability in relation to the control after 72 h of stimulus^37^.

*P. gingivalis* W83 strains have been known to adhere, invade and persist in bacteria-infected HCAEC^33^; however, its ability to activate endothelial cells by chemokine and cytokine production seems to exhibit slight activation to IL-8, IL-6, and MCP-1 compared to cells infected with other strains, such as 381 or 33277^33^. On the other hand, this study demonstrated inflammatory effect of W83 strain on endothelial cells, since HCAEC exposed to repeated exposure of live-*P. gingivalis* induced significant increases in chemokine levels, such as IL-8 and MCP-1, and in cytokines, such as IL-6, compared with the control group, while HCAEC cells challenged with a single exposure of *P. gingivalis* W83 showed similar production of IL-8, IL-6 and MCP-1, compared with uninfected cells.

Increases in IL-8 are involved in the firm adhesion of rolling monocytes in the early stages of atherogenesis^38^. Similarly, IL-6 and MCP-1 increases have been implicated in the adhesion of leukocytes (mainly monocytes) to activated endothelium, which contributes to cellular migration^39^. Experimental findings suggest differential efficacy of *P. gingivalis* to activate HCAEC cells. *P. gingivalis* 381 also enhanced IL-6, IL-8 and MCP-1 production and even the adhesion of immune cells with bacteria-infected HCAEC, while other *P. gingivalis* strains, such as W83, induced slight activation^33,40,41^.

*P. gingivalis*-LPS was a poor inducer of IL-6, MCP-1, GM-CSF and IL-8 from HCAECs supernatants, similar results has been shown in immune cells as dendritic cell (DCs)^42^. In contrast, several authors have shown that endotoxin of *P. gingivalis* strain 33277 induces an important pro-inflammatory effect, increasing the production of IL-8, soluble E-selectin and MCP-1 in HCAEC^43^, while 381 strains only exerted a weak stimulatory effect on HCAEC^41^. These results have been previously described and can be attributed to the presence of the K1 capsule or the characteristics of thechemotype of *P. gingivalis-LPS* W83 that may present differences in the O antigen region^44–46^, which leads to the structural variations of the LPS that can be related with attenuation of the immune system of the host. Therefore, the low inflammatory response in HCAEC cells challenged with LPS could not be generalized to all strains of *P. gingivalis*^16,33^.

It has been described that *P. gingivalis*-LPS is an inducer of tolerance in macrophages, mainly by the suppression of the endothelial recognition receptor (TLR-4)^47,48^; however, our results showed that LPS (7.0 µg/mL) induced an increase in the release of IL-8 at repeated exposures, which may be related to the type of endothelial recognition receptor to which *P. gingivalis*-LPS W83 binds (TLR-2) or other factors that may be involved by positively regulating the release of IL-8^49,50^. However, new studies are required to elucidate the mechanism of action.

Regarding the relationship between mRNA and proteins levels, cytokines as MCP-1 and IL-8 which were significantly detected in supernatants of HCAEC exposed to live-*P. gingivalis*, while MCP-1 mRNA levels were not upregulated. These discrepancies between mRNA and protein levels may involve the degradation rate of mRNA, which falls within a much tighter range (2–7 h for mammalian mRNAs vs 48 h for proteins)^51^. In fact, a previous report in aortic smooth muscle cells has determined that *in vitro* half-life of MCP-1 mRNA is approximately 45 min^52^, however, further verification using transcriptome analysis or RT-qPCR are required.

On the other hand, Ang II has been implied in atherogenesis promoting the oxidative stress in the vasculature, endothelial dysfunction and induction of an inflammatory response in the vessel wall^53^. Regardless endothelial cells are not considered a dominant source of AngII and AGTR^54^; previous studies have shown that classic LPS from enterobacterias stimulate local and circulatory Ang II levels^55–57^. However, knowledge regarding the *in vitro* vasoactive effects of periodontopathogens as *P. gingivalis*-LPS on HCAECs, the cell type that has typically been used in studies of atherosclerotic diseases, is unclear yet. Contrariwise, our data showed *P. gingivalis*-LPS as a poor gene inducer to Ang II, AGTR1, AGTR2 and all chemokine/cytokine measured. It also suggests a weak vasoactive and inflammatory effect of endotoxin isolated from *P. gingivalis* W83.

Interestingly, repeated exposure of live bacteria *P. gingivalis* induces a greatest effect in HCAEC, suggesting that Ang II can modulate signaling cascades associated with the release of pro-inflammatory cytokines through calcium mobilization, arachidonic acid production, kinases activation (MAPKs, PKC, JAK, PI3K) or the activation of transcription factors as cAMP and NF-Kβ^17,58–60^, which may explain the possible effects presented by the stimulation with live-*P. gingivalis* on endothelial cells^21,55^. However, further research is required to clarify possible mechanism.

Regarding the association between Ang II and the release of proinflammatory molecules such as IL8, IL6 and MCP1, there is no evidence with periodontopathogens, while some evidence have been reported *Escheriria coli-*LPS (*E. coli-LPS*)^48,58,59^, suggesting a synergistic effect between live-*P. gingivalis* stimulus and Ang II.

In contrast to the high concentration of Ang II at repeated dose of live bacteria, we found a down regulation of AGTR1 and AGTR2 at the mRNA level. Similar results with human saphenous vein cells (VSMC) and rat aortic smooth muscle (HASMC) suggest that high concentrations of Ang II can induce *in vitro* gen downregulation or desensitization/internationalization of AGTR1^48,58,59^ or AGTR2^57^. However, additional studies are required to identify the role of these receptors in endothelial dysfunction at repeated dose of periodontopathogens.

This work represents an alternative to the traditional *in-vitro* approach to evaluate *P. gingivalis* effects on endothelial cells, since transient and frequent bacteremia or endotoxemia episodes have been clearly described in patients with periodontitis. However, the classic stimulation model performed at a single dose up to 24 h did not expose the endothelium to these immunological challenges. Therefore, *in-vitro* exposure to more than one (repeated) exposure of *P. gingivalis* on the endothelium could lead to a better understanding for the study of endothelial dysfunction and pro-inflammatory activation.

## Materials and Methods

### Bacterial culture and inoculum standardization

*P. gingivalis* (BAA-308/W83) strain was obtained from the American Type Culture Collection (ATCC) and cultured using standard methods. This strain was originally isolated from humans with oral infections (i.e., periodontitis) and has been shown to be highly virulent compared with other *P. gingivalis* strains^60^. Bacteria were grown in supplemented Brucella agar (0.3% Bacto agar, 0.2% yeast extract, 5% defibrinated sheep blood, 0.2% haemolyzed blood, 0.0005% hemin, and 0.00005% menadione) and incubated at 37 °C for 4 days in anaerobic conditions (Anaerogen, Oxoid, Hampshire, UK)^61^. Bacterial inoculums were prepared and standardized for *P. gingivalis* in RPMI-1640 (Thermo Scientific, Waltham, MA, USA) and were quantified by spectrophotometry (Thermo Scientific, Waltham, MA, USA) at specific optical densities (OD) of 0.900–0.908 at a wavelength of 620 nm, corresponding to 2,6 × 10^9^ bacteria/mL. The count of colony forming units (CFU) was confirmed in triplicate under incubation conditions. Viable bacteria experiments were performed in a maximum time of two hours after having counted them, in order to avoid bacterial mortality.

### LPS extraction and purification

LPS extraction was performed using hot phenol-water, as previously reported^32^, with 1.1 g of wetted *P. gingivalis* W83; the purification was accomplished using an enzymatic treatment with nucleases and protease, followed by size-exclusion chromatography (Sephacryl S-200 HR) with sodium deoxycholate as the mobile phase^62^. The characterization of the LPS was determined by SDS-PAGE electrophoresis, purpald assay and chromogenic LAL test, compared to the commercial LPS of *P. gingivalis* ATCC 33277 (InvivoGen)^32^.

### Stimulation of HCAEC with LPS and viable bacteria in a single and repeated exposure model

HCAEC cells (LONZA, Walkersville USA) were cultured in supplemented EGM2 MV medium (LONZA, Walkersville, USA). The cells were used at passage 7 in growth medium (2 × 10^5^ cells/well) using 12-well culture plates (CytoOne, USA Scientific, Orlando FL, USA) and pre-incubated at 37 °C in a water-saturated atmosphere of 95% air and 5% CO_2_ until reaching confluence at 20 h. Subsequently, the cells were exposed to two treatment models at different concentrations of purified LPS (1.0, 3.5 and 7.0 µg/mL) and serial dilutions of live bacteria (MOI 1:100 - 1:0,1). A pilot study was conducted using longer exposure times where HCAEC cells were stimulated with P. gingivalis LPS on days 1, day 3 and day 5; the supernatant is removed every 48 hours to reach a total of 7 days under repeated exposure. The supernatant was stored at −80 C, for subsequent cytokine analysis.

In the first model the cells were stimulated for 6 h, after that, the stimulus was removed between each stimulus and replaced with a next exposure for another 6 h and for the last stimulation HCAEC the cells were exposed to another 12 h, for a total exposure time from 24 h. The supernatant was collected and storage for soluble factors measuring.

For the second treatment model, the conventional stimulus was referenced by literature in which HCAECs were stimulated with LPS at 1 µg/mL and  serial dilutions of live bacteria MOI (1:100 - 1:0,1)  at a single exposure for 24 h^43^.

### Cell viability assay

The cells were cultured in 96-well plates and stimulated according to the model described above with *P. gingvalis*-LPS (1.0, 3.5 and 7.0 µg/mL) and the live bacterium MOI 1:100. Subsequently to determine the cell viability was determined according to the fluorometric detection after the reduction of resazurin in the resorufin product using AlamarBlue (Biosource, Camarillo, CA, USA)^63^, the cells were placed in the medium containing 10% Alamar blue, after 10 h of incubation, 100 μL of the medium was transferred to the wells of a 96-well plate and the changes in the fluorescence with a microplate fluorometer equipped with an excitation filter set of 560 nm/590 nm emission (Infinite 200 PRO, Tecan, Männedorf, Switzerland). The unstimulated samples were considered our survival control, while those treated with 1% triton for 10 minutes were defined as positive control of death.

### RNA extraction and RT-qPCR

The mRNA expression levels of AGTR1, AGTR2, IL-8, IL-1β, MCP-1 and GAPDH were obtained from HCAEC cell stimulated to single or repeated exposures with LPS or live bacteria by qPCR. Total RNA was obtained from HCAEC using the QuickPrep MicroPrep isolation kit (Zymo Research, Irvine, CA, USA). The total amount of RNA was quantified using a Nanodrop (Thermo Scientific, Waltham, MA, USA). RT-qPCR was performed using 40 ng of total RNA and a RT-qPCR Luna Universal One-Step kit (New England Biolabs, Ipswich, MA, USA). Primers were designed using Beacon Designer software and are listed in Table 1. The reaction mixture consisted of 4,1 µL of template [10 ng/µL], 5 µL of Luna Universal One-Step Reaction Mix [2x], 0,5 µL of Luna WarmStart RT Enzyme Mix [20x], and 0,2 µL of primers [10 µM], in a final reaction volume of 10 µL. The temperature profile used was as follows: 55 °C for 10 min, 1 cycle at 95 °C for 1 min, followed by 40 cycles of amplification at 95 °C for 10 s and 60 °C for 30 min. Expression levels were calculated from the qPCR results based on the modified 2−ΔΔCt method suggested by Pfaffl^64^. For these calculations, GAPDH and unstimulated cells were used as controls.

**Table 1:** Primer sequences used for gene expression analysis by qPCR.

Gene	Primer Forward	Primer Reverse
AGTR1	5′-TCAGCCAGCGTCAGTTTCAA-3′	5′-GCCAGCAGCCAAATGATGATG-3′
AGTR2	5′-GACAGACCAAACATATAAGAAGGA-3′	5′-TCAGCTTGCTTAGTGCCTA-3′
COX-2	5′-GATGATGTATGCCACAATCT-3′	5′-AGTCTCTCCTATCAGTATTAGC-3′
IL-8	5′-TGTGAAGGTGCAGTTTTGCCAAGG-3′	5′-GTTGGCGCAGTGTGGTCCACTC-3′
IL-1β	5′-CTTTGAAGCTGATGGCCCTAAA-3′	5′-AGTGGTGGTCGGAGATTCGT-3′
MCP-1	5′-GAAAGTCTCTGCCGCCCTT-3′	5′- TTGATTGCATCTGGCTGAGCG-3′
GAPDH	5′-GGTGGTCTCCTCTGACTTCAACA-3′	5′-GTTGCTGTAGCCAAATTCGTTGT-3′

### Determination of chemokine and cytokine levels by flow cytometry

IL-6, MCP-1, IL-8, IL-1β, and GM-CSF levels were measured in culture supernatants from HCAEC stimulated with LPS or live bacteria by flow cytometry using a Human Proinflammatory Chemokine Panel (BioLegend, San Diego, CA, USA), according to the manufacturer’s instructions. The minimal detectable concentrations of IL-8, MCP-1, IL-6, IL-1β and GM-CSF were 1.9, 2.2, 3.6, 2.6 and 2.0 pg/mL, respectively. Flow cytometry was performed using the BD Accuri C6 flow cytometer (Becton Dickinson Biosciences, San Jose, CA, USA), and the data were processed using BD Accuri C6 Software.

### Angiotensin II concentration

Ang II levels were determined from the supernatants of the cultured HCAEC stimulated with LPS or live bacteria, using the EIA ELISA Kit (Cayman Chemical, Ann Arbor, MI, USA). To detect Ang II in the supernatants, sample extraction and ELISA were performed according to the manufacturer’s instructions^65^.

### Statistical analysis

All experiments were repeated at least 3 times for qPCR and flow cytometry. All data were expressed as the mean ± SEM. ELISA results were performed at least 3 times in duplicate. One-way variance analysis (ANOVA) and Tukey’s *post hoc* tests were performed for all analyses. A p-value < 0.05 was considered statistically significant.

## Conclusions

Repeated exposure live-*P. gingivalis* induces a greater pro-inflammatory response than single exposure, described by IL-8, IL-6, MCP-1 and GM-CS in HCAEC. The expression of AGTR1 and production of Ang II induced by live-*P. gingivalis* W83 showed the vasomotor effect of whole bacteria in HCAEC more than LPS. The findings of this study suggest that repeated exposure of *P. gingivalis* in HCAEC induces the activation of proinflammatory and vasoconstrictor molecules that lead to endothelial dysfunction as a key mechanism of the onset and progression of arterial hypertension (HT) and atherosclerosis, which requires more research.

## Supplementary information

Supplementary Information
