# Supplementary material for: Repeated Porphyromonas gingivalis W83 exposure leads to release pro-inflammatory cytokynes and angiotensin II in coronary artery endothelial cells
Source: Sci Rep. 2019 Dec 18;9:19379. doi: 10.1038/s41598-019-54259-y (PMC6920421; doi:10.1038/s41598-019-54259-y)
Supplement: Supplementary file 1 — Supplementary Information [file 41598_2019_54259_MOESM1_ESM.pdf]

Title Page

Repeated *Porphyromonas gingivalis* W83 exposure leads to release pro-inflammatory cytokynes and angiotensin II in coronary artery endothelial cells.

Sergio Viafara Garcia[1], Sandra Johanna Morantes[1], Yersson Chacon Quintero[1], Diana Marcela Castillo[1], Gloria Inés Lafaurie[1], Diana Marcela Buitrago[1*]

[1] Unit of Basic Oral Investigation-UIBO, School of Dentistry, Universidad El Bosque, Bogota, Colombia.

* Corresponding author: Diana M. Buitrago Ramirez, Unit of Basic Oral investigation-UIBO, Laboratory of Biotechnology, School of Dentistry, Universidad El Bosque. 110121 Bogotá D.C Colombia. Cra. 9 No. 131 A – 02, PBX (571) 6489000 ext. 1158.

Supplementary Information

Supplementary Figure 1. Comparison of IL8 secreted in HCAEC stimulated with *P. gingivalis-LPS* in the repeated exposure model at times of 24h and 7 days. Monolayers of HCAEC cultured in 12-well plates were stimulated with *P. gingivalis*-LPS (1.0, 3.5, 7.0 µg/mL) for 24 h and 7 days under repeated exposure (+++). After stimulation, levels of the following chemokine IL-8 were measured in cell culture supernatants using a cytometric. Symbol (*) means $p<0.05$ vs control cells; (**). Three independent experiments were performed; the results are presented as the means ± SEM (n=3).

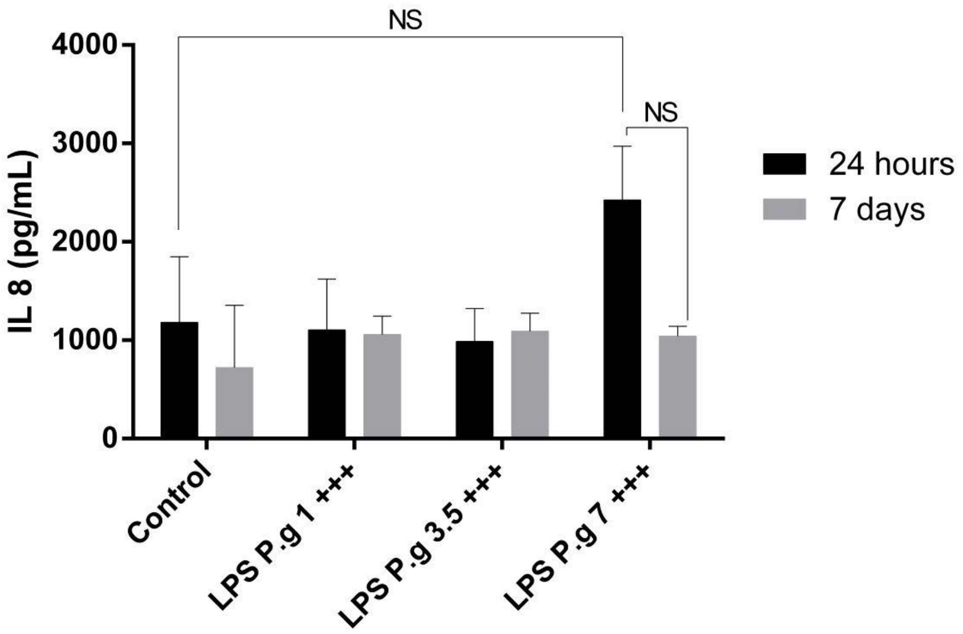

Supplementary Figure 2. cytokines secreted in HCAEC stimulated with *P. gingivalis*-LPS in the 7-day repeated exposure prolonged time model. Monolayers of HCAEC cultured in 12-well plates were stimulated with *P. gingivalis*-LPS (1.0, 3.5, 7.0 µg/mL) for 7 days under repeated exposure (+++). After stimulation, levels of the following chemokines were measured in cell culture supernatants using a cytometric bead array: (A) IL-6, (B) IL-1β. Symbol (*) means $p<0.05$ vs control cells. Three independent experiments were performed; the results are presented as the means ± SEM (n=2).

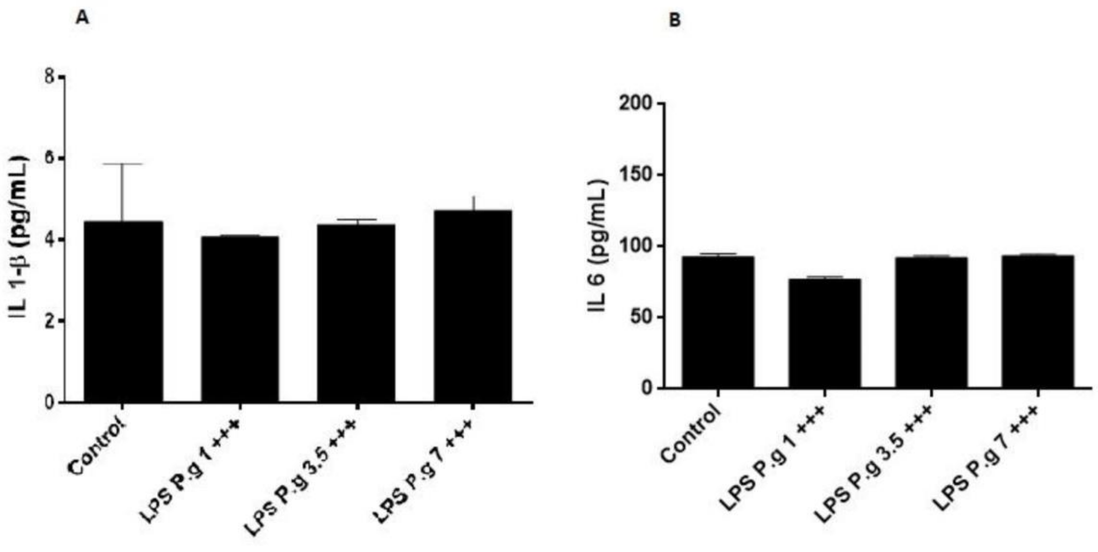
A
B
IL 1-β (pg/mL)
8
6
4
2
0
Control
LPS P.g 1 +++
LPS P.g 3.5 +++
LPS P.g 7 +++
IL 6 (pg/mL)
200
150
100
50
0
Control
LPS P.g 1 +++
LPS P.g 3.5 +++
LPS P.g 7 +++
